# Tendency to acidosis or alkalosis: Which one is associated with coronary artery disease?

**DOI:** 10.34172/jcvtr.025.33519

**Published:** 2025-12-17

**Authors:** Alireza Amirzadegan, Elahia Mohseni, Hassan Aghajani, Arash Jalali, Ahmad Vakili-Basir, Yeganeh Karimi

**Affiliations:** ^1^Tehran Heart Center, Cardiovascular Diseases Research Institute, Tehran University of Medical Sciences, Tehran, Iran; ^2^Department of Epidemiology and Biostatistics, School of Public Health, Tehran University of Medical Sciences, Tehran, Iran

**Keywords:** Coronary artery disease, Gensini score, Base excess

## Abstract

**Introduction:**

Base excess (BE) is an indicator of non-respiratory acid-base imbalances, which can impact coronary artery disease (CAD). This study evaluated the association between the severity of CAD and peripheral blood BE.

**Methods:**

This cross-sectional study included patients aged 18 and older who were candidates for coronary angiography. Demographic and clinical data were collected from medical records. Blood gas analysis was performed on a 2-millilitre arterial blood sample taken from the access artery before contrast injection. All patients underwent coronary angiography, and the Gensini score was calculated.

**Results:**

A total of 351 patients (194 males, 55.3%) were included in the study. The study population had a mean age of 60.79±9.5 and a mean BMI of 29.4±4.85. Coronary angiography revealed normal or minimal (<50% stenosis) findings in 51.3% of cases (15.4% with normal coronary arteries and 35.9% with minimal non-obstructive lesions), single-vessel disease in 17.4%, two-vessel disease in 14.5%, and three-vessel disease in 16.8%. Median Gensini score was 13.0, with an IQR of 3.5 and 49. The findings indicated that a decrease in BE was significantly correlated with elevated Gensini scores (β: -0.04; 95% CI: -0.08 to -0.01; *P*=0.027). However, BE did not significantly affect the Gensini score of 0 (*P*=0.843). Moreover, negative values of BE were significantly and inversely associated with the Gensini score (β=-0.05; 95% CI: -0.07 to -0.02, *P*<0.001).

**Conclusion:**

This study revealed an association between BE and CAD, suggesting that BE tending to acidosis is potentially associated with CAD.

## Introduction

 Atherosclerosis is still a leading cause of morbidity and mortality worldwide.^1^ Atherosclerotic-related diseases, such as ischemic heart disease (IHD), ischemic stroke, and peripheral artery disease (PAD), have caused significant mortality and disability-adjusted life years (DALYs) between 1990 and 2019, imposing a heavier burden on healthcare systems.^1^ In 2017, 126 million individuals were suffering from IHD, and nine million deaths were attributed to IHD.^2^

 The underlying cause of atherosclerosis involves inflammation and oxidative stress resulting from endothelial dysfunction, which then further exacerbates vascular endothelial dysfunction.^3,4^ While the precise mechanisms behind coronary atherosclerosis remain elusive, various risk factors have been associated with its development.^5-7^ For instance, metabolic acidosis has been recognized as an independent risk factor for arterial stiffness and the onset of peripheral vascular disease in individuals undergoing peritoneal dialysis.^8^ Identifying such risk factors contributes to our understanding and informs preventive and therapeutic approaches.

 The impact of intracellular pH on cellular function is well-established, highlighting the importance of maintaining a narrow pH range to optimize cellular function.^9,10^ Normal body fluid pH typically ranges from 7.35 to 7.45, maintained by the buffering effects of CO_2_ and HCO_3_.^11,12^ However, even among healthy individuals within this normal pH range, low-grade acidosis or alkalosis is prevalent.^12^ This state can be identified by measurement of base excess (BE) with a normal range of -2 to 2.^13^ BE quantifies the necessary amount of acid or base to normalize pH to 7.4, given specific conditions (PCO2 = 5.33 kPa or 40 mmHg) and a certain haemoglobin level.^14^ Thus, a BE less than zero indicates a tendency toward acidosis, while a BE greater than zero suggests a tendency toward alkalosis.^14^

 Evidence indicates that diet significantly impacts the pH and BE, consequently.^15^ Diets based on animal origin lead to the release of acid precursors, while plant-based diets lead to the release of base precursors.^16,17^ In addition, the literature shows the effect of diet on atherosclerosis and the risk of coronary vascular disease (CVD) by comparing a Western-type diet and a Mediterranean-type diet.^18^ Therefore, we hypothesize a potential link between BE, which can show a tendency towards acidosis, and CAD. The Gensini score, introduced by Gensini G. G., is used to quantify the severity of coronary artery disease (CAD).^19^

 To date, the potential correlation between BE and coronary atherosclerosis has not been explored. Therefore, the primary aim of our study is to investigate the association between the severity of coronary artery atherosclerosis, as measured by the Gensini Score, and peripheral blood BE levels.

## Materials and Methods

 This cross-sectional study was conducted at Tehran Heart Center, Tehran, Iran, between May 2022 and May 2023. We included patients over 18 years old undergoing coronary angiography. Exclusion criteria were as follows: a history of coronary artery bypass grafting, due to altered coronary anatomy that may confound the calculation of the Gensini score; significant valvular heart disease, which can impair cardiac output and tissue perfusion, potentially leading to acid–base imbalances; metabolic disorders known to affect blood gas parameters, including chronic liver disease, end-stage renal disease (glomerular filtration rate (GFR) < 15 mL/min/1.73 m^2^), sepsis, and diabetic ketoacidosis; major dietary changes within the week prior to angiography (e.g., fasting or adherence to a ketogenic diet), which may acutely alter acid–base status; and patients undergoing primary percutaneous coronary intervention (PPCI), due to the acute ischemic and hemodynamic changes.

 We gathered demographic and clinical data from medical records, including comorbidities, family history of cardiovascular diseases, and smoking habits. Body mass index (BMI) was determined using the formula: weight in kilograms divided by height in square meters. The primary outcome of this study was the Gensini score, reflecting the presence and severity of atherosclerosis, and the main independent variable was BE, measured in blood gas analysis.

###  Laboratory Measurement

 Blood samples were collected from participants upon admission to assess lipid profiles, fasting blood glucose (FBS), and creatinine (Cr) levels. Additionally, a 2-millilitre arterial blood sample was taken from the access artery just before the injection of the contrast agent for blood gas analysis. The blood gas analysis was performed by a SIEMENS RAPID Point 500 blood gas analyzer.

###  Coronary Angiography and Gensini Score

 Coronary angiography was performed according to the routine protocol of the center by expert interventionists. In summary, after sterilizing the patient’s skin, the radial or femoral artery was accessed using Seldinger’s technique. The guiding catheter was inserted into the relevant coronary artery, and the contrast dye was injected. Two experienced cardiologists interpreted the angiograms.

 According to the American College of Cardiology/American Heart Association (ACC/AHA) guidelines, CAD was defined as over 50% stenosis in any major coronary artery.^20^ Patients were classified into four categories: normal or minimal coronary artery changes (including patients with minimal non-obstructive lesions), one-vessel disease, two-vessel disease, and three-vessel disease. The Gensini score was calculated to define quantitative CAD severity.^19^ The Gensini score was calculated based on the extent and degree of coronary artery stenosis in addition to classifying it as follows: 1 point for 1-25% stenosis, 2 points for 26-50%, 4 points for 51-75%, 8 points for 76-90%, 16 points for 91-99%, and 32 points for complete occlusion. This score was then multiplied by a factor depending on the occlusion location, with different values assigned to various segments of the coronary arteries. The score would be multiplied by five if the stenosis was localized in the left main coronary artery, 2.5 if it localized in proximal left anterior descending (LAD) and left circumflex (LCX), 1.5 if it localized in the mid-segment LAD and LCX, one if it localized in the distal segment of LAD and LCX, first diagonal branch, first obtuse marginal branch, right coronary artery, posterior descending artery and intermediate arteries, and 0.5 if it localized in the second diagonal and second obtuse marginal branches.

###  Sample size calculation 

 Considering that 15% of angiography results are normal at our center, 342 consecutive cases are required at a significance level of 0.05 (α = 0.05) and a study power of at least 80% to detect an odds ratio (OR) of 1.5 (or its reverse as 0.67) for the association between BE and a positive Gensini score compared to a Gensini score of 0 (reflecting the presence and severity of coronary atherosclerosis).

###  Statistical Analysis 

 Categorical variables were presented as frequencies and percentages, while continuous variables were described using either the mean with standard deviation (SD) for normally distributed variables or the median with interquartile range boundaries (25th and 75th percentiles) for variables exhibiting skewness. The assessment of normality for these variables involved the examination of measures of central tendency, dispersion, and histogram charts. We employed the stabilized Inverse Probability Weighting (IPW) technique to adjust the effects of potential confounders identified through a comprehensive literature review on blood BE. Furthermore, we constructed a standardized mean difference (SMD) plot to visualize and assess the covariate balance graphically. Due to the excess zeros of the Gensini score (n = 54), to evaluate the effect of blood BE on the Gensini score, we use a two-part model considering sIPWs. In the two-part model, the effect of blood BE on Gensini = 0 is explained and interpreted using logistic regression (with reporting OR with 95% confidence interval (CI)), while for Gensini > 0 using linear regression with log-transform (reporting beta coefficient with 95%CI). Analyses were conducted using the R Statistical language (version 4.3.0; R),^21^ using the packages WeightIt (version 0.14.2)^22^ and twopartm (version 0.1.0).^23^

###  Ethics and consent

 We ensured written informed consent upon admission from all participants to participate in this study and use their clinical data anonymously for research purposes. The Tehran University of Medical Sciences’ research board and medical ethics committee approved the study (approval code: ir.tums.thc.rec.1400.080). The study was conducted in accordance with the Helsinki Declaration.

## Results

 The study included 351 patients (194 males, 55.3%) who underwent coronary angiography. Table 1 presents the baseline characteristics of the participants. The average age of the study group was 60.79 years, with an SD of 9.5 years. The majority of the patients were overweight, having a mean BMI of 29.4 with an SD of 4.85. Seventy-seven patients (24.9%) were current smokers and 43 (12.3%) patients used opioids. Dyslipidemia was observed in 82.9% of the participants. Diabetes mellitus and hypertension were prevalent in 43.9% and 60.4% of the participants, respectively. Patients’ average GFR was 88.20 ± 29.82 mL/min/1.73 m^2^. A family history of CAD was noted in 25.1% (88 individuals) of the study group. Blood gas analysis revealed the median and IQR values for blood pH, blood (bBE), and extracellular fluid BE as 7.4 (7.4, 7.4), 0.0 (-1.7, 1.3), and -0.2 (-2.4, 1.2), respectively. Results of coronary angiography showed that 51.3% of cases were either normal or had minimal changes in the coronary arteries (15.4% with normal coronary arteries and 35.9% with minimal non-obstructive lesions), 17.4% had single-vessel disease, 14.5% had two-vessel disease, and 16.8% had three-vessel disease. The overall median Gensini score was 13.0, with an IQR of 3.5 to 49.0. Patients with normal/minimal coronary angiography results had a median bBE of 0.00 (IQR: -1.50 to 1.30). In contrast, patients with CAD had a median bBE of -0.40 (IQR: -1.90 to 1.45).

**Table 1:** Baseline characteristics of study participants

**Demographic**		**Laboratory findings**		**Angiography results**	
**Characteristic**	**n=351**^a^	**Characteristic**	**n=351**^a^	**Characteristic**	**n=351**^a^
Gender (*Male*)	194 (55.3%)	ECF base excess (mmol/L)	-0.2 (-2.4, 1.2)	Gensini score	13.0 (3.5,49)
Age (*year*)	60.79 ± 9.50	Blood base excess (mmol/L)	0.0 (-1.7, 1.3)	CAG result	
BMI (*kg/m*^2^)	29.40 ± 4.85	Serum HCO3 (mmol/L)	23.94 ± 3.22	*Normal/Minimal*	180 (51.3%)
Opium ever use	43 (12.3%)	Serum PCO2 (kPa)	4.78 ± 0.73	*SVD*	61 (17.4%)
Current smoker	77 (21.9%)	Serum PH	7.4 (7.4, 7.4)	*2VD*	51 (14.5%)
Hypertension	212 (60.4%)	Serum creatinine (umol/L)	79.58 (70.74, 97.26)	*3VD*	59 (16.8%)
Dyslipidemia	291 (82.9%)	TG (mmol/L)	1.52 (1.1, 2.05)		
Diabetes mellitus	154 (43.9%)	TCH (mmol/L)	3.94 ± 1.12		
History of CVA or TIA	21 (6.0%)	LDL (mmol/L)	2.17 (1.71, 2.59)		
Family history of CAD	88 (25.1%)	HDL (mmol/L)	1.12 ± 0.3		
		FBS (mmol/L)	5.99 (5.27, 7.59)		
		GFR (mL/min/1.73 m^2^)	88.20 ± 29.82		
		GFR < 60	54 (15.4%)		

^a^n (%); Mean ± SD; Median (IQR) CVA: Cerebrovascular accident; TIA: Transient ischemic accident; CAD: Coronary Artery Disease; ECF: Extracellular Fluid; LDL: Low-density lipoprotein; HDL: High-density lipoprotein; FBS: Fasting blood sugar; GFR: glomerular filtration rate; CAG: Coronary angiography; SVD: Single vessel

**Figure 1:**
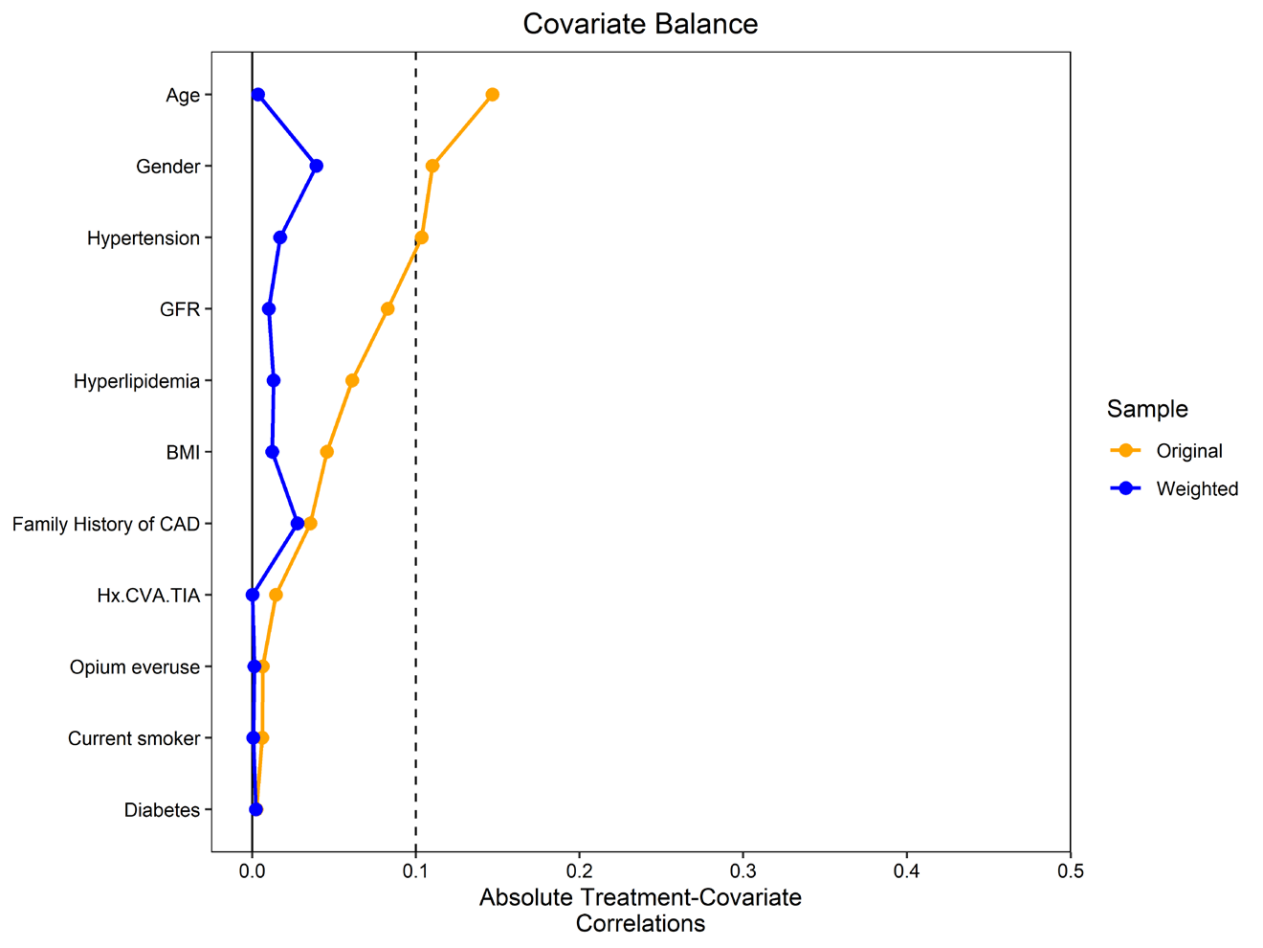
Standardized Mean Differences (SMD) for blood base excess.The standardized mean differences (SMD) before (Original) and after weighting (Weighted), are given as absolute values, for all covariates included in the model. The standardized difference compares the difference in means between groups in standard deviation units. After weighting, the differences between groups were < 10% (dashed line), showing good covariate balance (n = 351)

**Figure 2:**
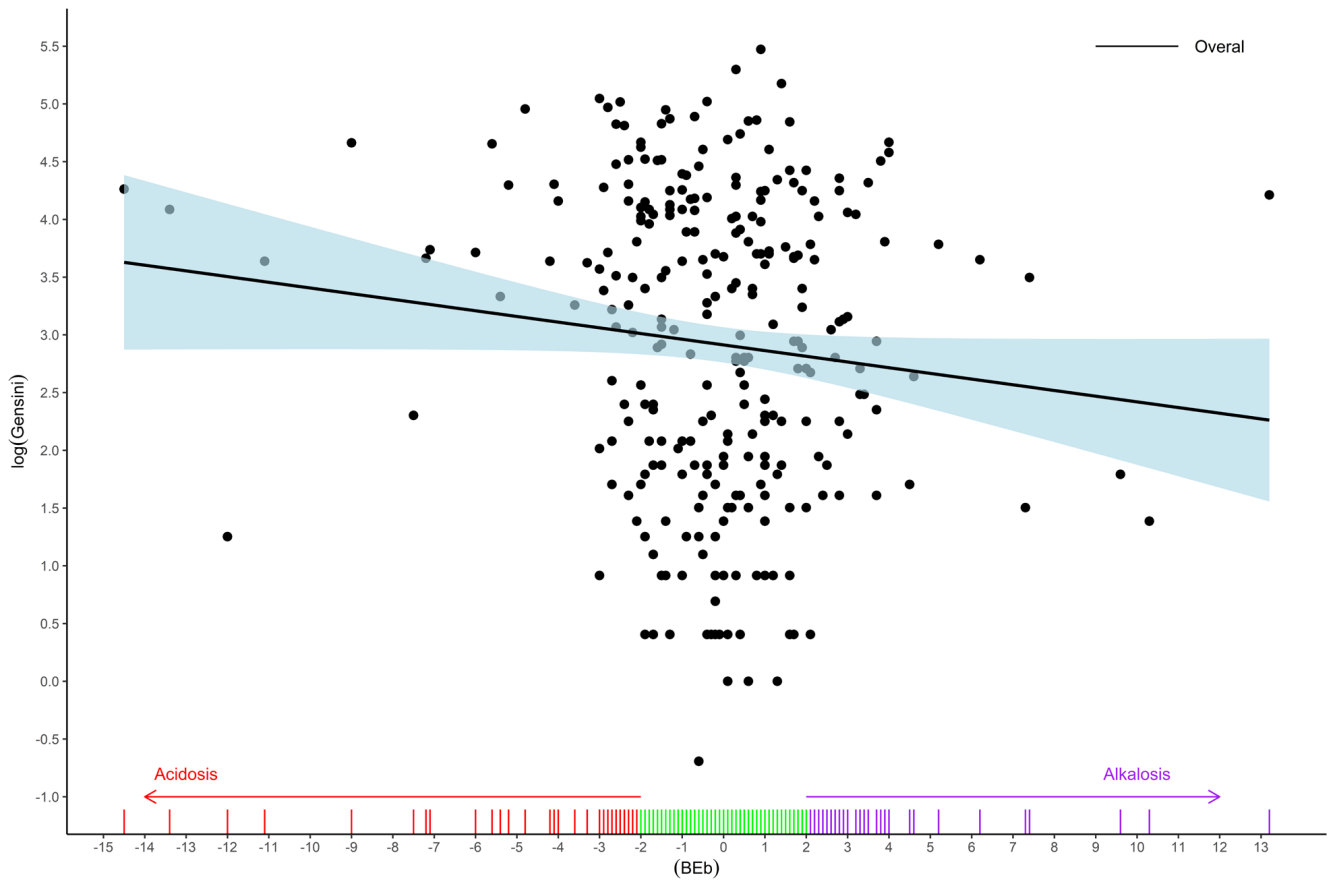
A plot of blood base excess against the logarithm of Gensini (n=351)

**Figure 3:**
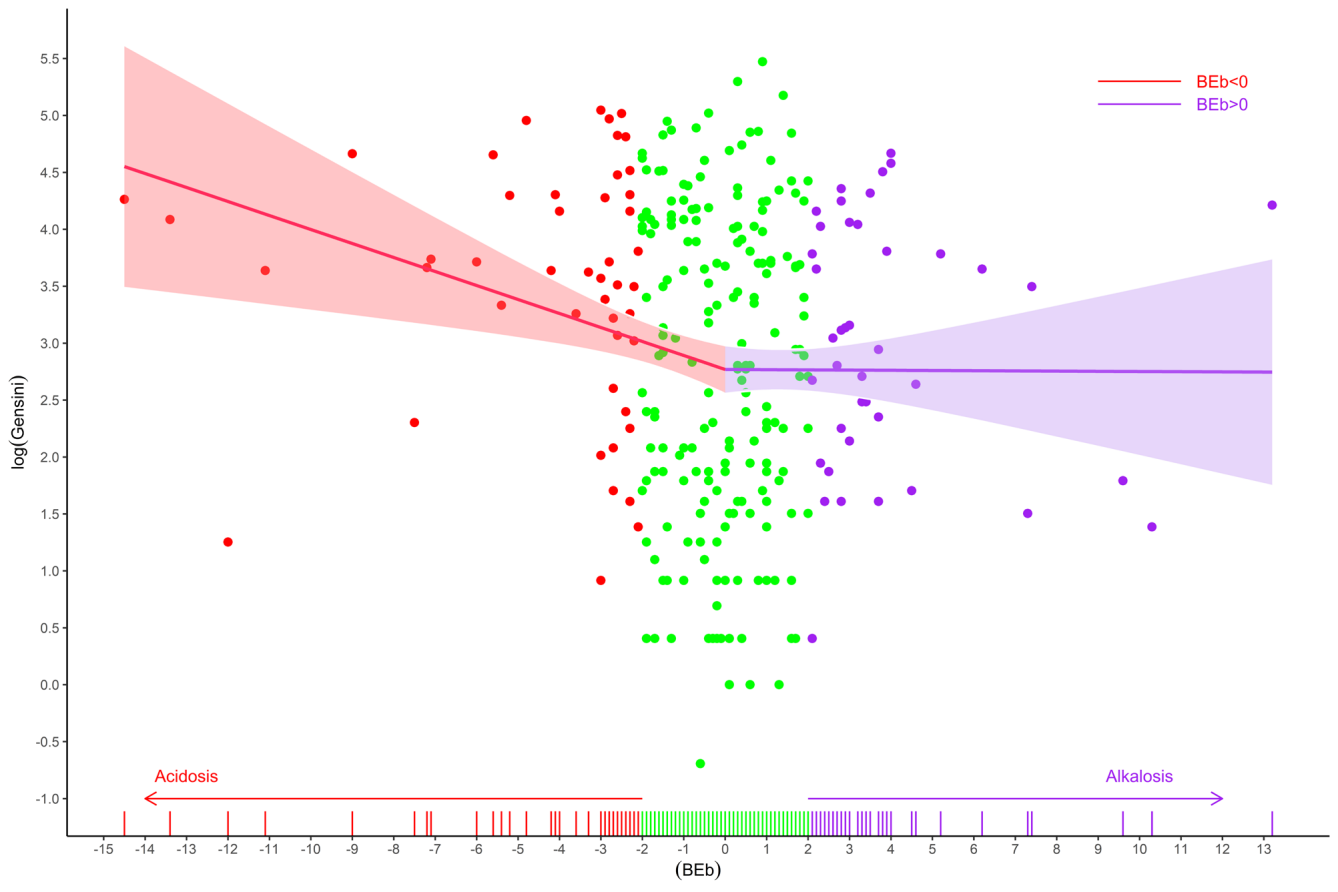
A plot of blood base excess against the logarithm of Gensini (n=351)

 To evaluate the relationship between BE and Gensini score, weighted values of BE were calculated considering age, gender, BMI, underlying diseases, GFR, past medical history, and smoking status as confounders. Figure 1 represents standardized mean differences for bBE. Given the frequent occurrence of a Gensini score of 0, a two-part model was used to examine the influence of BE on the Gensini score (refer to Table 2). The findings indicated that bBE did not significantly affect the Gensini score of 0 (*P* = 0.843). However, a decrease in bBE was significantly correlated with elevated Gensini scores, with a unit decrease in BE associated with a 0.04 increase in the logarithm of Gensini score (OR: -0.04; 95% CI: -0.08 to -0.01; *P* = 0.027). While both negative and positive bBE values inversely influenced the Gensini score, only the impact of negative values was statistically significant (β = -0.05; 95% CI: -0.07 to -0.02, *p* < 0.001). On the other hand, positive BE values did not significantly affect the Gensini score (β = -0.01; 95% CI: -0.01 to 0.09, *p* = 0.853). Figures 2 and 3 show the relationship between overall bBE and the separated positive or negative values of bBE and the Gensini score, indicating a strong effect of negative BE values on the increased Gensini score.

**Table 2:** Two-part model for weighted Blood base excess (bBE) effects on Gensini score (total sample size: 351, patients with Gensini score = 0: 54)

**Characteristic**	**Zero Part (Logistic regression)**			**Non-zero Part (linear regression with log-transform)**		
	**OR**	**95% CI**	***P*** ** value**	β ^a^	**95% CI**	***P*** ** value**
bBE	1.01	0.92, 1.11	0.843	-0.04	-0.08, -0.01	0.027
bBE^b^	0.94	0.84, 1.06	0.336	-0.05	-0.07, -0.02	< 0.001
bBE^c^	0.97	0.79, 1.20	0.820	-0.01	-0.10, 0.09	0.853

OR = Odds Ratio, CI = Confidence Interval, bBE = blood base excess ^a^ β = Effect on the logarithm of Gensini ^b^ Only negative values of BEb included in this analysis ^c^ Only positive values of BEb included in this analysis

## Discussion

 The findings of this study highlight the association between weighted BE levels and the severity of CAD, showing a reverse relationship between BE levels and the Gensini score. To the best of our knowledge, this is the first study to evaluate the association between BE levels and Gensini score.

 Our findings align with a previous study that indicates an association between BE levels and metabolic acidosis with cardiovascular diseases.^8^ Terlecki et al recently showed a significant negative correlation between BE levels and the Global Registry of Acute Coronary Events (GRACE) risk score and mortality rates at 30 and 365 days in myocardial infarction patients undergoing percutaneous coronary intervention.^24^ This finding is supported by evidence suggesting that negative BE serves as an independent predictor of mortality in the intensive coronary care unit (ICCU) during the initial phase of myocardial infarction.^25^ Additionally, reduced BE levels have been recognized as an independent risk factor for all-cause mortality in critically ill patients suffering from acute myocardial infarction.^26^ In contrast, Nakano et al reported that elevated BE was associated with higher long-term mortality in patients with acute heart failure.^27^ Raikou et al found an inverse relationship between serum bicarbonate levels and arterial stiffness indicators, indicating that metabolic acidosis is an independent risk factor for arterial stiffening and peripheral vascular disease in the context of renal failure and peritoneal dialysis.^8^ This is corroborated by another study that discussed the association between metabolic acidosis and cardiovascular diseases.^28^

 The association between BE levels and atherosclerosis can be explained by the role of metabolic acidosis in promoting vascular inflammation and oxidative stress.^29,30^ A decline in BE is indicative of a tendency toward metabolic acidosis. Additionally, several studies have shown that older individuals might experience low-grade acidosis, which can be indicated by BE.^31,32^ Wurzinger et al found significant correlations between oxidative stress markers and metabolic stress indicators, such as peroxidase activity and oxidation lag time, with pH, HCO3, and BE.^30^ The association between oxidative stress, inflammation, and endothelial dysfunction leading to atherosclerosis was further explained by Higashi et al.^4^ Additionally, the impact of metabolic acidosis on vascular calcification through disturbances in mineral metabolism has been observed in dialysis patients, linking vascular calcification with increased cardiovascular events.^33,34^ Interestingly, a significant correlation was observed between BE and coagulation impairment in trauma patients upon emergency department arrival.^35-37^ As the decreased BE may lead to an increased Gensini score, decreased BE may be a consequence of myocardial ischemia, which strengthens the association between BE and CAD.^38^

 The clinical implications of these findings suggest that decreased BE might be a risk factor for CAD. Although there is no evidence representing the association between BE and CAD, as quantified by the Gensini score, several studies indicate the predictive value of BE in other conditions. Importantly, BE proved to be an independent predictor for both in-hospital and post-discharge mortality in patients with myocardial infarction.^24,26^ In addition, Guo et al reported a U-shaped relationship between BE levels and the risk of all‐cause death in patients with congestive heart failure (CHF).^39^ This finding indicates that both high and low BE values increased CHF mortality.^39^ Nakano H also found an association between high BE, but not low BE, and long-term mortality in acute heart failure patients.^27^ Another study identified decreased BE as an independent predictor of in-hospital mortality and organ malperfusion in acute type B aortic dissection patients.^40^

 This study revealed that patients exhibiting lower peripheral blood BE, indicative of a tendency towards acidosis within the normal pH range, tend to show more severe CAD, as assessed by the Gensini Score. However, higher levels of positive BE were not associated with the Gensini score. Consequently, these results suggest that decreased BE could serve as a potential risk factor for CAD, thus contributing to a deeper comprehension of the underlying mechanisms involved in atherosclerosis.

 This study examined the relationship between BE and the Gensini score, with the calculation of weighted BE enhancing the study’s strength. Limitations include its cross-sectional design, which does not establish causality between BE and CAD severity, and the potential lack of generalizability due to the single-center study population. Due to the impact of dietary habits on BE, the precise evaluation of nutritional habits is another limitation of this study. Moreover, BE may have been influenced by unrecognized confounding factors. Addressing these limitations in future studies will enhance the validity and generalizability of findings.

## Conclusion

 In conclusion, the study suggests a potential association between negative BE and the severity of CAD, as evidenced by the correlation between lower BE values and higher Gensini scores. Further research is necessary to validate this correlation. Therefore, interventions aimed at increasing BE, such as adopting plant-based diets, may be considered preventive strategies against CAD.

## Competing Interests

 The authors report no conflicts of interest.

## Ethical Approval

 All participants gave written informed consent to participate in this study and anonymously share their clinical data for research purposes. The Tehran University of Medical Sciences’ research board and medical ethics committee approved the study (approval code: ir.tums.thc.rec.1400.080).
